# Porous Au-embedded WO_3_ Nanowire Structure for Efficient Detection of CH_4_ and H_2_S

**DOI:** 10.1038/srep11040

**Published:** 2015-06-18

**Authors:** Nguyen Minh Vuong, Dojin Kim, Hyojin Kim

**Affiliations:** 1 Department of Materials Science and Engineering, Chungnam National University, Daejeon, 305-764 Republic of Korea; 2 Department of Physics, Quy Nhon University, 170 An Duong Vuong, Quy Nhon, Binh Dinh, Vietnam

## Abstract

We developed a facile method to fabricate highly porous Au-embedded WO_3_ nanowire structures for efficient sensing of CH_4_ and H_2_S gases. Highly porous single-wall carbon nanotubes were used as template to fabricate WO_3_ nanowire structures with high porosity. Gold nanoparticles were decorated on the tungsten nanowires by dipping in HAuCl_4_ solution, followed by oxidation. The surface morphology, structure, and electrical properties of the fabricated WO_3_ and Au-embedded WO_3_ nanowire structures were examined by scanning electron microscopy, transmission electron microscopy, X-ray diffraction, X-ray photoelectron spectroscopy, and current–voltage measurements. Formation of a nanowire structure resulted in significant enhancement in sensing response to H_2_S and CH_4_ gases. Furthermore, Au embedment into the WO_3_ nanowire structures remarkably improved the performance of the sensors. The increase in response performance of sensors and adsorption–desorption kinetic processes on the sensing layers were discussed in relation with the role of Au embedment.

Gas sensors based on semiconducting metal oxides have been reported as indispensable devices that can be used in different daily applications. Compared with many other oxide materials, gas sensors based on tungsten oxide (WO_3_), which is an n-type semiconductor because of oxygen vacancies^1^, have attracted much attention because of the inherent electrical resistivity, excellent response, and selectivity of WO_3_ to various gases. Sensing performance, particularly the response of a sensor, can be controlled by modifying the properties of sensing layer, including grain size, porosity, thickness, morphology, and impurities, by surface modification with novel metals^2^. Surface modification with catalytic novel metals particularly showed important advantages. Reactivity of a material surface and solid–gas interactions can be controlled by using various modifiers at different concentrations. For example, the configuration of d electrons of transition metals on the surface of a material can change the surface activity of the oxide semiconductor^3^,^4^,^5^.

Metal nanoparticle decoration and doping of WO_3_ nanostructures have been widely studied, and the modified materials showed desirable properties, thereby improving the performance of devices. Dopants modify the electronic structure by forming impurity levels in bandgaps; dopants are employed to enhance the responses of a sensor to specific gases^6^,^7^,^8^,^9^. In addition, decoration of metal nanoparticles on WO_3_ surface may lead to the formation of rectifying Schottky junctions at the interfaces to alter electrical transport behavior of WO_3_ and/or activate gas molecules that react with WO_3_ surface. For example, Pt and Pd nanoparticles enhance the dissociation of H_2_ molecules on WO_3_ surface via the known spillover effect, thereby improving the performance of H_2_ gas sensor and gaschromic devices^10^,^11^.

In this study, we report a facile method to embed gold catalyst on highly porous WO_3_ nanowires. The high-porosity nanowire structures of Au-embedded WO_3_were synthesized by using porous single-wall carbon nanotubes (SWCNTs) as template, followed by tungsten deposition, dip coating in gold salt solution, and oxidation. We examined the effects of Au-embedment on structural, electrical, and gas sensing properties of the fabricated sensor. Sensors based on nanowire structures of WO_3_ showed a significant improvement in sensing performance towards CH_4_ and H_2_S gases. In particular, WO_3_-based materials for CH_4_ sensors have not been reported. However, the additional embedment of Au further enhanced the sensing performance. We found that the Au-embedded WO_3_ sensor showed high response to CH_4_. Embedding of Au nanoparticles on WO_3_ surface also showed interesting response properties toward H_2_S gas. The adsorption–desorption kinetic processes on the surface of Au nanoparticles and WO_3_ nanowires surface were also discussed.

## Results

### Structural Properties

**Figure 1:**
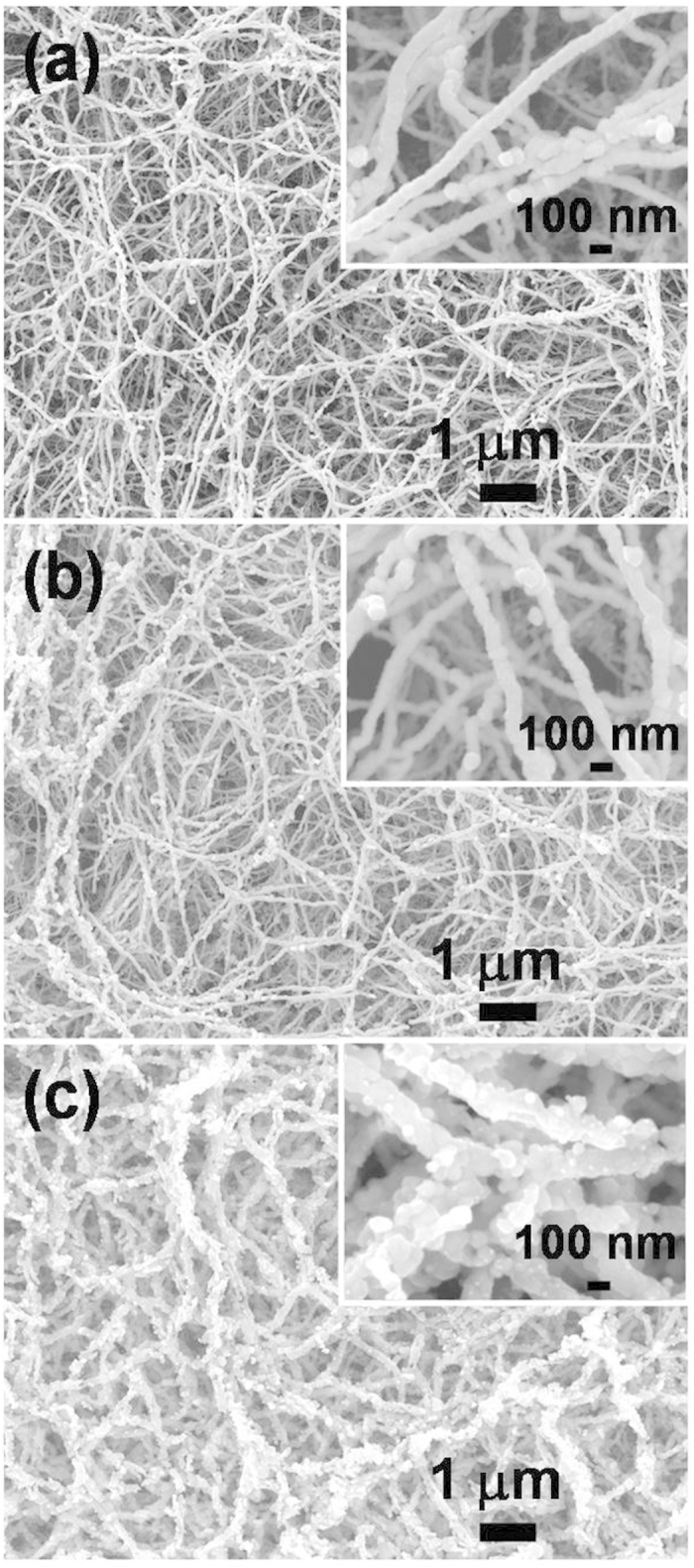
Morphology of the (**a**) as-synthesized tungsten-SWCNT nanowires, (**b**) tungsten-SWCNT nanowires immerged in HAuCl_4_ solution. and (**c**) Au-embedded WO_3_ nanowire structure fabricated by oxidation of (**b**) at 700 °C. Inset images show high-magnification SEM.

**Figure 2:**
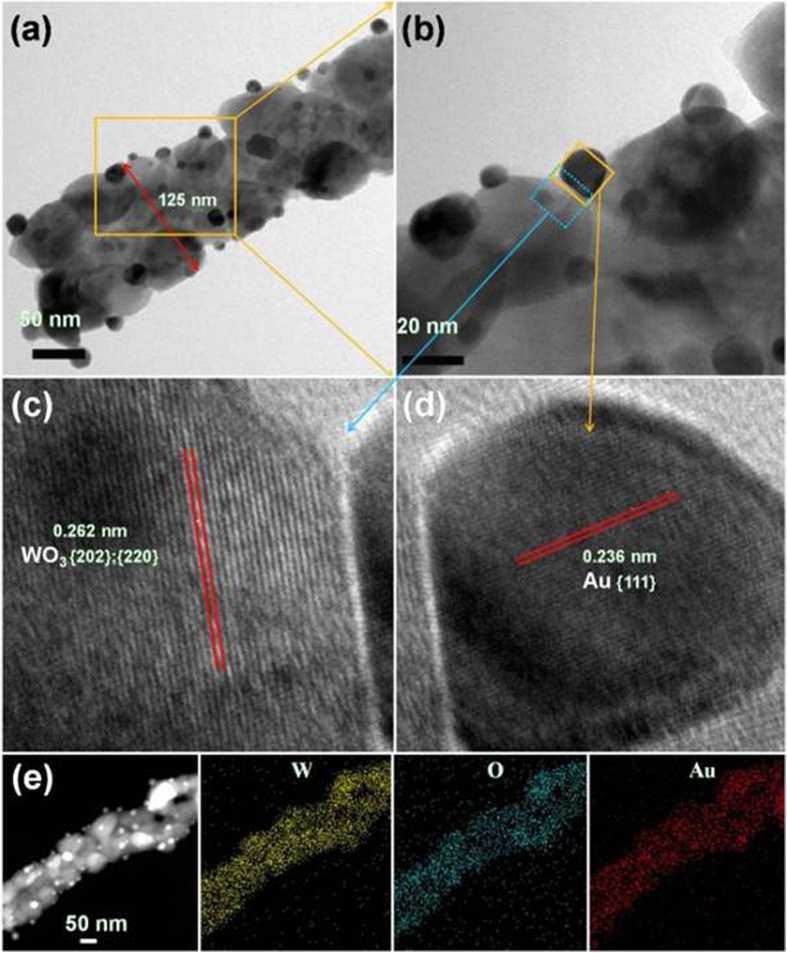
(**a,b**) TEM and (**c,d**) HR-TEM images of the Au-embedded WO3 nanowire structure. (**e**) STEM images and the corresponding STEM–EDX elemental mapping of the Au-embedded WO_3_ nanowire structure.

**Figure 3:**
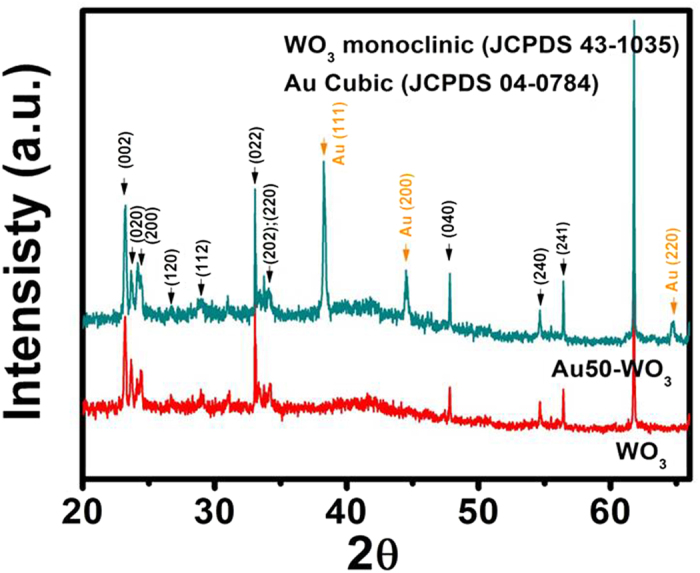
XRD patterns of WO_3_ and Au-embedded WO_3_ nanowire structures.

Porous nanowires of tungsten were obtained by sputter deposition of tungsten metal on porous SWCNTs template (Fig. 1a). The characteristic nature of the open spaces of the SWCNT template was stable during the metal sputter deposition and the following oxidation processes, as previously reported^12^. The morphology was negligibly changed after immersion in Au-containing solution (Fig. 1b and Fig. S3). As seen in Fig. 1c, WO_3_ nanowire structure was formed by interconnection of WO_3_ grains during the oxidation process at 700 °C in air. Gold atoms on the surface also agglomerated to Au nanoparticles during the oxidation process. The distribution of Au nanoparticles was non-continuous and scattered on the surface, as revealed by the low-magnification TEM images in Fig. 2a,b. The diameter of the gold nanoparticles ranges from 8 nm to 20 nm and the average diameter of WO_3_ nanowires is ~120 nm. The HR-TEM images revealed a high crystallinity of the synthesized WO_3_ nanowires and gold particles. The interplanar spacing of *d*_{202{220}_ = 0.262 nm in Fig. 2c is consistent with the monoclinic phase of WO_3_ [JCPDF file no. 43-1035], whereas *d*_{111}_ = 0.236 nm in Fig. 2d correspond to the cubic phase of Au [JCPDF file no. 04–0784]. The XRD pattern of the WO_3_ and Au50-WO_3_ nanowire structures (Fig.3) also confirmed the formation of monoclinic phase WO_3_ structure [JCPDF file no. 43-1035] and cubic phase of Au structure [JCPDF file no. 04-0784]. The bottom images show elemental mapping for W, O, and Au of the Au-embedded WO_3_ nanowire. The images were obtained by STEM–energy-dispersive X-ray (EDX) spectroscopy, which suggest that gold atoms not only form nanoparticles on the WO_3_ nanowire surface, but also diffuse inside of the WO_3_ crystalline lattice.

**Figure 4:**
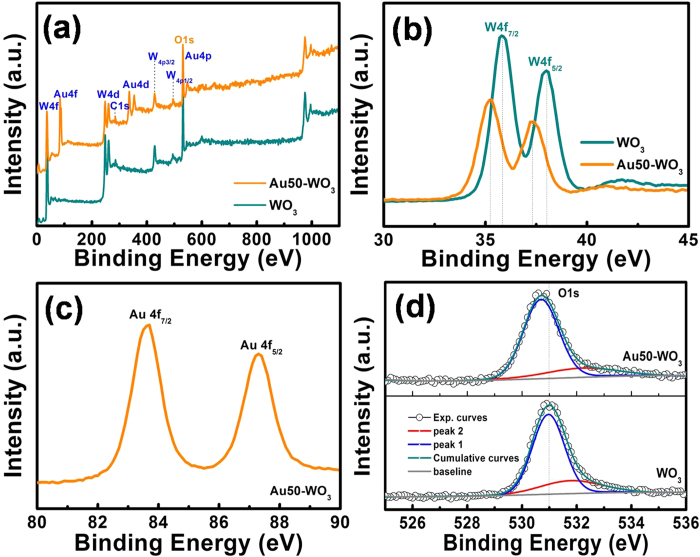
(**a**) XPS spectra of WO_3_ and Au-embedded WO_3_ nanowire structures. (**b–d**) High-resolution spectrum of W, Au, and O peaks.

To investigate the surface composition and chemical states of the elements in the sample, XPS spectra of the nanowire structures of WO_3_ and Au50-WO_3_ were obtained. The spectrum in Fig. 4a indicates that the surface of the samples is composed of tungsten and oxygen elements with carbon contamination. In addition, Au distribution after annealing of Au-embedded WO_3_ sample is evident. The peak at 285.7 eV is attributed to CO_2_, which is commonly adsorbed on the surface of the samples^13^. The high-resolution spectra of W4f (Fig. 4b) showed peaks at 35.85 and 38.02 eV, which are assigned to W4f_7/2_ and W4f_5/2_, respectively. These peaks corresponded to oxidation state +6 of tungsten atoms (WO_3_)^14^,^15^. The shift of these peaks toward lower binding energy in Au50-WO_3_ can be attributed to photoelectrons emitted from the lower oxidation states of tungsten (sub-stoichiometric WO_3−x_)^14^. The Au4f core level spectrum (Fig. 4c) recorded from the Au50-WO_3_ sample showed peaks at 83.6 and 87.3 eV, which were assigned to Au4f_7/2_ and Au4f_5/2_, respectively. These data confirmed that the Au nanoparticles are in metallic state^15^. The O1s peak in Fig. 4d showed a shift to lower binding energy with Au-embedment suggesting increased defective or incomplete W–O binding via Au addition. The O1s peak could be resolved to two Gaussian function peaks (1) and (2) having binding energies of 531 and 531.8 eV for WO_3_ nanowire structure, and 530.7 and 532.2 eV for Au50-WO_3_ nanowires, respectively. Peak (1) may be related to oxygen in the lattice and O^2−^ ions, and peak (2) might be due to the ions of O^2−^, O^−^, and OH^−^ in the oxygen-deficient regions^16^. A shift of the peak (1) to lower binding energy and a shift of the peak (2) to higher binding energy were observed with Au embedment. The shift of the major peak (1) to lower binding energy with Au embedment may be attributed to the defective WO_3_ or sub-stoichiometry of WO_3−x_, whereas the shift of peak (2) may suggest an increased binding energy of the ionosorbed oxygen via Au addition. Details of the causes for the latter observation or the stronger binding of the oxygen ions on the surface are unknown, but it is consistent with the delayed surface reaction kinetics in Au50-WO_3_, which will be discussed.

### DC conductivity measurements

**Figure 5:**
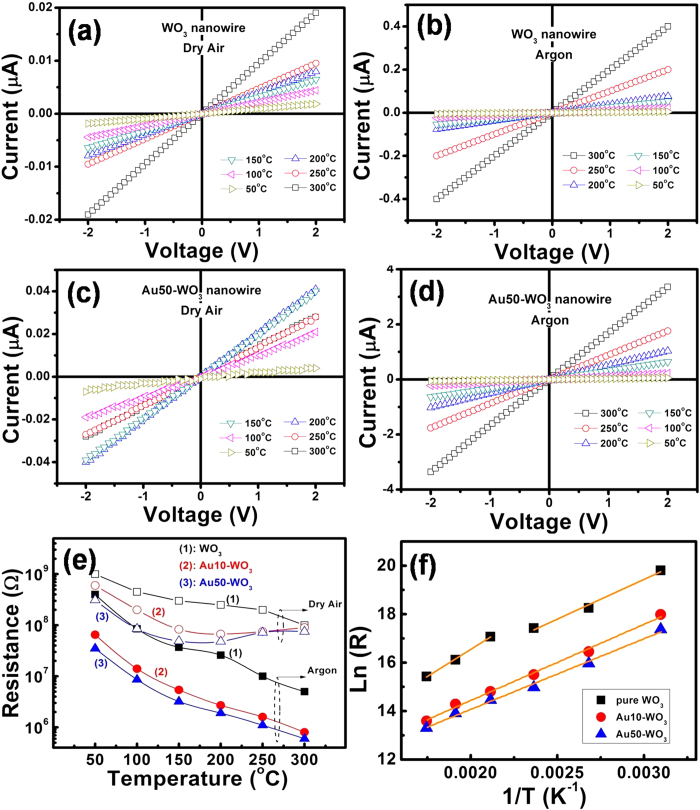
The current–voltage characteristics of (**a,b**) the WO3 nanowire sensor measured in dry air and argon ambient conditions, and (**c, d**) the Au-embedded WO3 nanowire sensor in dry air and argon ambient conditions. (**e**) Dependence of the resistance of the WO_3_ and Au-embedded WO_3_ nanowire sensors on different working temperatures. (**f**) The relationship between ln(R) and 1/T for the WO_3_ and Au-embedded WO_3_ nanowire structures in the argon condition (the straight lines show the fitting).

The current–voltage (I–V) characteristics of the fabricated nanowires devices, as fundamental properties of electronic materials, were measured with varying temperatures in argon and dry air. Prior to the I–V measurements, the devices were heated to 350 °C in the given ambient conditions to remove the effect of adsorbed water molecules on the surface of the nanowires. The I–V curves of both WO_3_ and Au50-WO_3_ are linear, as shown in Fig. 5a-d, indicating the formation of ohmic contacts between the nanowires and Au electrodes. The resistances of nanowires calculated from the I–V measurements for pure WO_3_, Au10-WO_3_, and Au50-WO_3_ nanowire structures are shown in Fig. 5e. The measurements in Ar ambient condition are not affected by oxygen ionosorption effect; results revealed monotonic decrease of the resistance with increasing temperature for all sensors. This relationship indicates a dominant semiconducting behavior. Therefore, the metallic nature of Au did not alter the semiconducting resistance–temperature behavior of the WO_3_ nanowires probably because of the discreteness of the distribution of Au particles on the surface (Fig. 2). Nevertheless, a finite Au-embedment effect was observed, in which the resistance decreased with increasing content of embedded Au.

The increase in conduction with Au embedment can be explained by the Au-doping effect in the WO_3_ lattice. This possibility could be postulated based on the EDS data in Fig. 2, which shows a uniform distribution of Au solid through the WO_3_ nanowire body. Although most of the Au atoms agglomerated on the surface as nano-crystallites, some atoms are incorporated into the WO_3_ nanowires during the annealing process and might form small Au clusters in theWO_3_ lattice. If the conductivity of a transition metal oxide is established by hopping of electrons via dopant impurity levels, then the resistance of the oxide can be expressed by the following equation^17^,^18^:


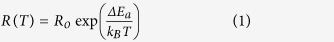


where *R(T)* is the resistance of a semiconductor at the absolute temperature *T*, *ΔE*_*a*_ is the thermal activation energy for hopping, and *k*_*B*_ is the Boltzmann constant. The temperature-dependent resistances of the pure WO_*3*_ and Au-embedded WO_*3*_ nanowire structures are shown in Fig. 5f, which were obtained by using the logarithm of equation (1). For pure WO_3_ nanowires structure, we estimated the activation energies in two temperature ranges because of the slight anomaly observed at 200 °C. The activation energy values were ~0.39 eV for 200–300 °C, and ~0.29 eV for 50–150 °C. These values are all greater than the thermal activation energies of 0.27 and 0.25 eV observed for Au10-WO_3_and Au50-WO_3_ nanowire structures, respectively. The decrease in activation energies with increasing Au concentration might be due to creation of additional impurities in the lattice. The distribution of Au clusters in the WO_3_ lattice could somehow increase the oxygen vacancies. Some Au atoms may have occupied the lattice sites to play directly as a dopant. Au atoms can create impurities in the bandgap, but the observation is in agreement with the cases of In-doped CdS^19^ and Al- and Sb-doped CdTe^20^, which showed a decrease in activation energy with increasing dopant concentrations. In summary, the increase in conductance with Au embedment was analyzed, but a concrete conclusion cannot be elucidated at present and separate systematic study is necessary.

Interestingly, Fig. 5e shows that the temperature-dependent resistance behaviors of the Au-embedded WO_3_ nanowires in air deviated from the semiconducting behavior at temperatures >150 °C; the resistances increased with increasing temperature. The increase in resistance is due to an enhanced oxygen ionosorption rate and corresponding increase of surface depletion depth of WO_3_ nanowires. This phenomenon is the catalytic effect of the embedded Au nanoparticles because the pure WO_3_ did not show such deviation even in air environment. Activation of oxygen adsorption on the surface of tin oxide decorated with various novel metals has been observed previously^21^. We found that the catalytic effect of Au critically enhances the dissociation of oxygen molecules at >150 °C, at which the adsorption ionic form of oxygen changes from O_2_^−^ to O^−^22.

### Gas sensing properties

#### *CH*_*4*_ *gas sensing*

**Figure 6:**
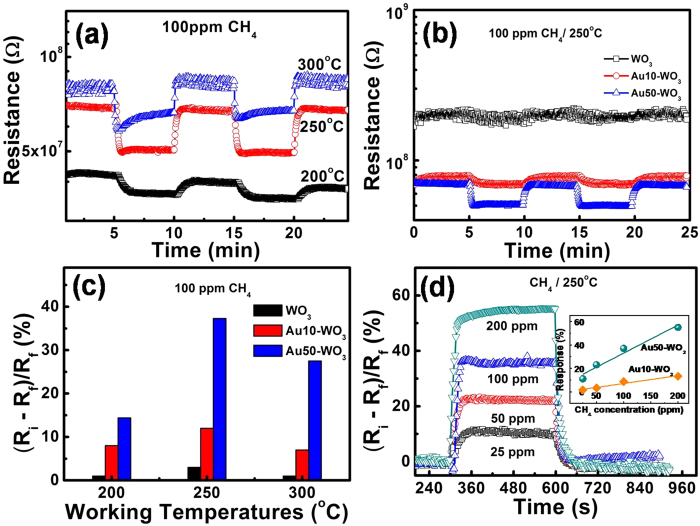
(**a**) Gas-sensing properties of Au50-WO_3_ nanowire sensors upon exposure to 100 ppm CH_4_ at different working temperatures. (**b**) Gas-sensing properties of pure WO_3_ and Au-embedded WO_3_ nanowire structures at optimal working temperature of 250 °C upon exposure to 100 ppm CH_4_. (**c**) The responses of sensors to 100 ppm CH_4_ at different working temperatures were summarized. (**d**) The response of Au50-WO_3_ nanowire sensor to various CH_4_ gas concentrations at optimal working temperature of 250 °C and its summary showing linearity in sensing CH_4_ concentration (inset).

The development of resistive sensors based on metal oxide semiconductors for CH_4_ detection has been given much research interests in recent years. Among the metal oxides, tin oxide (SnO_2_) semiconductors have attracted particular attention^2^, but sensing by WO_3_ have been seldom reported. The CH_4_ gas sensing properties of pure WO_3_ and Au-embedded WO_3_ nanowire sensors were examined and compared. The sensors exhibited a typical gas-sensing behavior of an n-type semiconductor, as shown by the decrease in sensor resistance with exposure to the reducing gas CH_4_. The response and recovery behaviours of the Au50-WO_3_ nanowire sensor upon exposure to 100 ppm CH_4_ diluted in dry air was measured at different operating temperatures of 200 °C, 250 °C, and 300 °C, as shown in Fig. 6a. The response behaviours of WO_3_, Au10-WO_3_, and Au50-WO_3_ sensors measured at the optimum working temperature of 250 °C are compared in Fig. 6b. The trends of temperature dependence of the sensor signal were the same in all the sensors, showing the highest response at 250 °C. The increasing Au content revealed increasing response signals, as shown in Fig. 6c. The response with pure WO_3_ nanowire sensor was very low, which may be the reason for the limited reports on CH_4_ gas sensing by WO_3_-based sensor. The response enhancement was from 3% with pure WO_3_ to 37% with Au50-WO_3_ sensor at 100 ppm CH_4_ and 250 °C. The sensing behaviors of Au50-WO_3_ sensor for different CH_4_ gas concentrations measured at 250 °C are shown in Fig. 6d. The concentration dependence of the responses for Au10-WO_3_ and Au50-WO_3_ are shown as the inset. Notably, the Au50-WO_3_ sensor showed a reduced operating temperature in comparison with the SnO_2_-based methane sensors, as shown in our previous report^23^.

The enhanced response with Au embedment is ascribed to the catalytic effect of Au on the sensing process. We described the gas adsorption and desorption kinetics in relation to gas sensing of semiconducting WO_3_ nanowires, and found that the response level and the gas selectivity of an oxide sensor is derived from the ratio of the forward (adsorption) reaction rate over the reverse (desorption) reaction rate of the relevant gas species on the oxide surface^24^,^25^. The higher response in the Au-embedded structure indicates that the reaction rate of CH_4_ with ionosorbed oxygen was enhanced with respect to the recovery rate, which is determined by the oxygen adsorption rate in air. We observed that Au has a catalytic effect on oxygen ionosorption and enhances the adsorption rate at >150 °C, which will enhance the recovery rate above. However, given that the enhanced response with Au-embedded sensor dictates far higher enhancement in the reaction CH_4_+ 4O^−^→CO_2_+2H_2_O+4e^−^, gold has to further accelerate the CH_4_ decomposition reaction. For example, for methane dissociated to a methyl group and a hydrogen adatom on Au surface^26^, the dissociated molecular species can more actively react with the adsorbed atomic oxygen than the covalently bonded CH_4_ molecules. In conclusion, Au showed catalytic effects on CH_4_ dissociation and oxygen adsorption, but the rate-enhancement level was greater with CH_4_ than with O_2_. The net consequence was the enhanced sensor response for CH_4_ with Au-embedded WO_3_ sensor structures.

We also reported that a rush of reducing gas, such as H_2_ and NH_3_, on oxygen-ionosorbed oxide surface can produce an instant pile up of H_2_O molecules on the surface, leading to an overshoot in the response cycle curves^24^,^25^. The similar overshoots in the response cycles were observed with CH_4_ on Au50-WO_3_sensor at 250–300 °C, as shown in Fig. 6a. We believe the overshoots were also derived from the combined effect of the enhanced reaction rate at high temperature and the high impingement rate of C, H, and other fractions of CH_4_ catalytically dissociated by Au.

#### *H*_*2*_ *S gas sensing*

**Figure 7:**
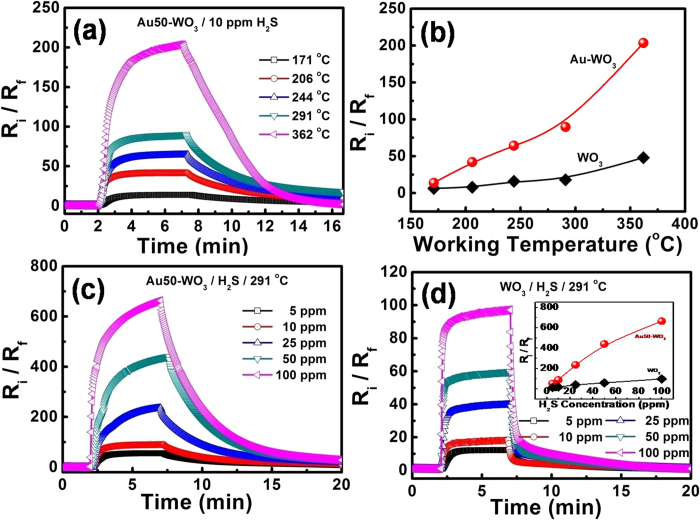
(**a**) Gas-sensing properties of Au50-WO_3_ nanowire sensors upon exposure to 10 ppm H_2_S at different working temperatures. (**b**) Summary of (**a**) together with the measurements from WO_3_ nanowire sensor. The response of (**c**) Au50-WO3 and (**d**) pure WO_3_ nanowire sensors to various H_2_S gas concentrations at optimal working temperature of 291 °C. Inset shows the summary.

**Figure 8:**
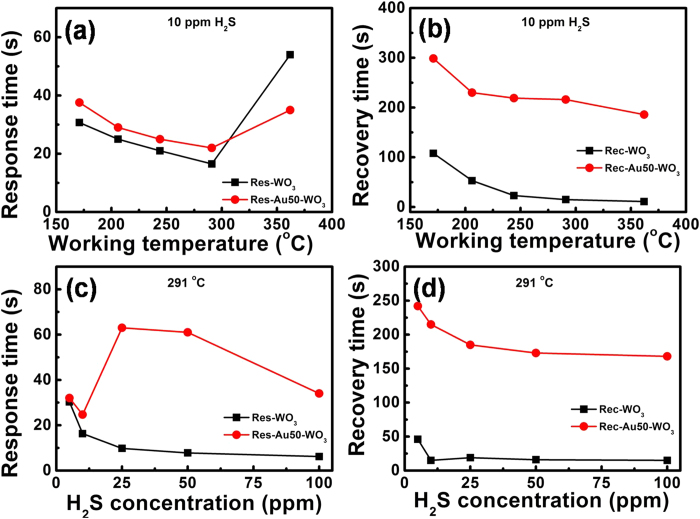
The response and recovery time of WO_3_ and Au50-WO3 nanowire sensors measured (**a,b**) at different working temperatures to10 pm H_2_S gas concentration, and (**c,d**) at optimal working temperatures of 291 °C on various H_2_S gas concentrations.

The operation temperature effect of H_2_S gas sensing was examined for pure WO_3_ and Au50-WO_3_. Fig. 7a shows the response-and-recovery curves of Au50-WO_3_ nanowire sensor upon exposure to 10 ppm H_2_S at different operating temperatures (171–362 °C). The response levels are summarized in Fig. 7b. Remarkable improvement in the responses by Au-embedment was observed at all the operation temperatures up to 362 °C. The sudden increase of the response times at 362 °C in both sensors was interesting (Fig. 8a). This phenomenon may be due to the catalytic decomposition of H_2_S occurring in temperatures between 300 °C and 400 °C^27^,^28^. Thus, the species binding on the sensing layer changes at ~300 °C. Therefore, the operating temperature was set at 291 °C for the sensing of H_2_S to distinguish it from the different sensing mechanisms at >300 °C.

The response behaviors of Au50-WO_3_ and WO_3_ nanowire sensors to H_2_S were measured at various concentrations of 5, 10, 25, 50, and 100 ppm diluted in dry air at operating temperature of 291 °C, as shown in Figs. 7c, d. The responses of these sensors at different gas concentrations were also summarized in Fig. 7d, as shown in the inset. A response of 700 was obtained with the Au50-WO_3_ nanowire sensor at 100 ppm H_2_S (Fig. 7c), which is much higher than the response of 100 obtained from the pure WO_3_ nanowire sensor (Fig. 7d). This result can definitely be explained by the catalytic effect of gold. The catalytically fractionized H_2_S species could more actively react with ionosorbed oxygen. The WO_3_ and Au50-WO_3_ nanowire sensors also showed excellent selectivity to H_2_S, as is evident from Fig. S4. The response to H_2_S was more than two orders of magnitude higher than the response to other interfering gases, including H_2_, NH_3_, CO and CH_4_ the same concentration. The selectivity towards H_2_S was further enhanced by the Au embedding (Fig. S4).

However, the Au50-WO_3_ nanowires showed much slower response and recovery rates compared with the pure WO_3_ nanowires through the whole working temperatures <300 °C and the tested H_2_S gas concentrations, as summarized in Fig. 8. Herein, the response and recovery times were measured assuming exponential rise and decay of the curves based on the first-order surface reaction kinetics for adsorption and desorption^24^,^25^. Therefore, they are the characteristic average times of the processes and are the times required for completion of approximately 63% (1-1/e) of the response and recovery processes. The decrease in response and recovery times with increasing working temperatures (Figs. 8a,b) is basically due to the enhanced surface chemical reaction rates in the response and recovery cycles. An exception is the abnormal increase of the response time at 362 °C with both pure WO_3_ and Au50-WO_3_ nanowire structures caused by the change in the formula of the species adsorbing on the Au and WO_3_ surfaces. Its effect on WO_3_ conductance will be discussed.

The response and recovery times measured with varying H_2_S concentrations are shown in Figs. 8c,d. The decreasing response time with increasing gas concentrations observed with the pure WO_3_ nanowires is the general trend in the sensing of reducing gases when the sensing behavior is based on a simple chemical reaction on the surface^24^,^25^. However, interestingly, the response time abnormally changes with H_2_S concentration for the Au-embedded sample, as shown in Fig. 8c. The result reveals a difference in H_2_S gas-sensing mechanism between pure WO_3_ and Au-embedded WO_3_ structures.

Generally, the response levels were enhanced but the response-recovery kinetics slowed down with Au-embedment. The improved gas-sensing response levels were definitely assisted by the Au nanoparticles. As we systematically examined in Refs. 25 and 29, the response and recovery processes of the WO_3_ sensing layer towards reducing gases are the results of thermally activated chemical reaction processes on the surface. H_2_S gas molecules continuously react with the pre-absorbed oxygen ions (say O^−^)^22^ via


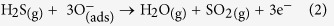


to form H_2_O and SO_2_. Meanwhile, oxygen molecules from the air environment continuously adsorb on the empty adsorption sites on the surface in the air atmosphere. Therefore, the response level to H_2_S reflects the steady-state distribution of oxygen ionosorption on the WO_3_ nanowire surface under continuous impingement of H_2_S and O_2_molecules. In addition, creation of additional surface oxygen vacancies^29^ due to


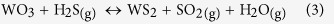


sequent reduction of W^6+^ to W^4+^to release electrons into the WO_3_ nanowires was also proposed to be the reason for the response. However, a study of the detailed chemical route for sensing is beyond the scope of the present research.

Therefore, the enhanced response in Au50-WO_3_ nanowire sensor with respect to the pure WO_3_ (Fig. 7b) can be attributed to the highly catalytic dissociation of H_2_S by Au nanoparticles. As can be recall from Fig. 5c, the Au nanoparticles also enhanced the dissociation rate for oxygen adsorption. Therefore, the higher response observed with Au nanoparticles than with the pure WO_3_ could be derived from the increase in desorption rate of the adsorbed oxygen ions in equation (2), which was propelled by both of the enhanced dissociation reactions of H_2_S and O_2_.

A probable explanation for the enhanced catalytic role of Au in increasing the response of the sensor may be derived from the literature. H_2_S is known to adsorb strongly onto Au because of the high chemical affinity of S on Au^30^. Leavitt and Beebe^31^ found that H_2_S decomposes to SH via


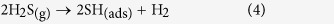


to chemisorb onto the Au surface, and the formed H_2_ is released in the temperature range of 165–520 K. At >520 K, the adsorbed SH undergoes disproportionation to form gaseous H_2_S and S-adsorption on the Au surface via


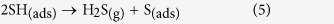


The binding of S on Au can lower the surface work function of Au as much as 1 eV^32^, so this lowering of Au work function would decrease the band bending at the interface of Au and WO_3_ nanowires, leading to a further resistance decrease and an improved response in the Au-embedded WO_3_ sensor. Both effects enhance the sensor response and explain the Au catalytic effect.

Now, the issues that need to be addressed are as follows: (i) the longer response and recovery times of Au-embedded WO_3_ than WO_3_ (Fig. 8), (ii) the abnormal increase of the response time at 362 °C(Fig. 8a), and (iii) the sudden rise of response time for higher H_2_S concentration in Au-embedded WO_3_ (Fig. 8c). The slower response and recovery kinetics with Au-embedment should be caused by some delayed reactions in relation to Au nanoparticles. A simple reaction of (2) may explain the sensing with pure WO_3_. The decreasing response time as the H_2_S concentration increased in Fig. 8c for WO_3_ reflects the concentration-dependent surface reaction rate (or 1/τ∝ C_H2S_), which is observed when the surface reaction kinetics controls the sensing^24^,^25^. By contrast, additional reactions, such as


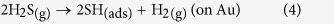



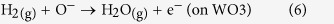


may occur with Au at relatively low temperatures. The locally generated H_2_ gas molecules are added to the WO_3_ surface for additional reactions. While this added surface reaction increased the sensor response level, the dissociation reaction on Au and/or the successive reaction on WO_3_ surfaces delayed the overall response and recovery processes. As the H_2_S concentration increased, further delayed responses were observed due to the absolute increase of the reactions, but the contribution of the reactions occurring directly on the WO_3_ surface [or equation (2)] became relatively dominant at far higher H_2_S concentrations, resulting in reduced response time (Fig. 8c).

For the recoveries, the simple re-adsorption of oxygen atoms on the WO_3_ surface explains the recovery cycle for WO_3_. However, for Au-embedded WO_3_, the surfaces not only of WO_3_, but also of Au particles, need to be recovered to the standby condition. In other words, chemical reactions, such as 2SH_(ads)_+O_2_→H_2_S_(g)_+SO_2(g)_ and/or S_(ads)_+O_2_→SO_2(g)_, are required to recover to the original Au surface condition. The reactions themselves require time and may be slow, and also can influence the processes occurring on nearby WO_3_ surface and finally determine the response time. For example, the produced H_2_S gas from the Au surface can impinge on the WO_3_ surface to play the role of analyte gas molecule and hinder the oxygen ionosorption. The delayed response and recovery rates are clear for different working temperatures and H_2_S gas concentrations, as shown in Fig. 8.

Another observation is that the further slowing down of the response rate at higher temperature of 362 °C was observed in both pure WO_3_ and Au-WO_3_ sensor structures (Fig. 8a). Therefore, it is a temperature-related reaction kinetics that occurs on WO_3_. A new reaction causing the apparently slower kinetics suddenly emerged at the above-mentioned temperature. The new reaction could be caused by the sudden change in (i) the formula of analyte gas provided and/or (ii) the ionosorbed oxygen formulas, resulting in a change in the reaction equation on the WO_3_ surface. If the impinging H_2_S gas molecules directly decompose to S and H_2_ and adsorb on the WO_3_ surface via equation (3), the high temperature further accelerates the decomposition reaction. The delayed escape of the highly increased product gas of H_2_O out of the surface could increase the apparent response time. Otherwise, the direct decomposition of H_2_S on the WO_3_ surface via


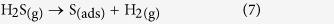


can be accelerated at the high temperature, thereby producing higher H_2_. The increased impingement of H_2_ on the surface could increase the apparent response time. The third possibility is due to the ionic form of adsorbed oxygen that may change from O^−^ to O^2–^ at such a high temperature [O_2_+4e→2O^2−^_(ads)_]^22^. The binding energy of O^2−^ is expected to be greater than that of O^–^; thus, the desorption of the former is more difficult. This situation led to the longer response time.

## Conclusions

Highly porous Au-embedded WO_3_ nanowire structures with diameters of ~120 nm were fabricated by tungsten deposition on porous SWCNT template, followed by dipping in HAuCl_4_ solution and oxidation. The Au atoms coated on the W nanowire surface agglomerated to form Au crystallites on the surface and in the WO_3_ lattice. The effects of Au, particularly of the embedded Au crystallites on the surface, on the gas-sensing properties were investigated based on the surface reactions. A comparison of the foregoing findings with the results obtained from pure WO_3_ nanowires is useful to discuss the Au embedment effect. For CH_4_ gas-sensing performance, the WO_3_ nanowire structure showed a significant lowering of working temperature with respect to SnO_2_ structure, whereas the Au-embedded structure demonstrated enhanced gas-sensing response because of improved dissociation of CH_4_ gas molecules. A different sensing performance of Au-embedded WO_3_ nanowires was observed for H_2_S gas. Although the gas-sensing signals were similarly improved, the response and recovery kinetics were slowed down by Au. Such complication seems to have originated from the diverse reaction routes of H_2_S gas on the Au and WO_3_ surfaces. The intermediate gas products on the surface may be impinged on the surface and play the role of analyte gas (such as H_2_) and/or temporarily adsorbed on the surface and show delayed detachment (such as H_2_O). The Au particles embedded on the surface catalyzed the dissociation reactions of O_2_, CH_4_, and H_2_S gases, thereby enhancing the oxygen ionosorption reaction and the gas-sensing reactions.

## Methods

### Fabrication of sensor structures

The Au patterned substrates were installed on the inside wall of the arc-discharge chamber; the substrate were then coated with SWCNTs, as described in Ref. 12 and 35.

Synthesis was performed at an arc current density of 40 A/cm^2^ in H_2_ gas at 400 Torr for 4 min. The carbon source used was a graphite rod that contained catalyst wires of iron, nickel, and molybdenum^12^,^33^,^34^. The SWCNT template substrates were heat-treated at 400 °C in air for 2 h to remove amorphous carbon. The SWCNT bundles seated on the substrate revealed large open spaces among the SWCNTs because of steric hindrance^12^. Tungsten metal layers were deposited on the SWCNT templates using a DC magnetron sputtering system. The deposition was performed at room temperature under a constant Ar pressure of 3.8 × 10^−2^ Torr and input power of 100 W for 120 s. During deposition, the substrates were rotated to attain uniform thickness. The conformal deposition of W along the porous SWCNT bundles also revealed highly porous W/SWCNT composite nanowires. Gold salt solutions were prepared by dissolving different amounts of HAuCl_4_ salt in the mixture of distilled water and ethanol at 1:3 ratio. The W/SWCNT samples were dip-coated with Au by immersing in gold salt solutions of 0, 10, and 50 mM of HAuCl_4_; the samples were identified as WO_3_, Au10-WO_3_, and Au50-WO_3_, respectively. Oxidation of the samples at 700 °C for 2 h resulted in conversion of W to WO_3_ and formation of Au nanoparticles while burning out the SWCNTs, leaving a highly porous tungsten oxide nanowire network. Fig.S1 shows the flowchart of the fabrication process for open-space ensemble Au-embedded WO_3_ nanowires structure using a highly porous SWCNT template.

### Characterization

Surface morphology of the nanowire structures was investigated by field emission scanning electron microscopy (FESEM; JEOL, JSM-700F) and transmission electron microscopy (TEM), scanning TEM (STEM), and high-resolution TEM (HR-TEM). The structural properties were investigated by X-ray diffraction (XRD; Rigaku D/MAX-RC) using Cu Kα radiation with a Ni filter. The electronic structure of the surface of samples was elucidated by X-ray photoelectron spectroscopy (XPS; VGMultilab 2000; Thermo VG Scientific, UK).

### Gas sensing property measurement

Gas sensing properties were also measured using 6487 Keithley in a chamber with controls for temperature and gas flow^12^,^35^. The response of a sensor, S, was defined by R_i_/R_f_ or (R_i_-R_f_)/R_f_ for reducing gases, where R_i_ is the standby resistance in air, and R_f_ is the resistance upon exposure to reducing gas. We used 1000 ppm CH_4_ and H_2_S gases diluted in nitrogen as the source gas, which was further diluted in dry air at varying concentrations in the ppm range. The structure of gas sensor is shown in Figs. S2a–c. The setup for the gas sensing measurements is shown in Fig. S2d.

## Additional Information

**How to cite this article**: Minh Vuong, N. *et al.* Porous Au-embedded WO_3_ Nanowire Structure for Efficient Detection of CH_4_ and H_2_S. *Sci. Rep.* **5**, 11040; doi: 10.1038/srep11040 (2015).

## Supplementary Material

Supplementary Information
