# Supplementary material for: Porous Au-embedded WO3 Nanowire Structure for Efficient Detection of CH4 and H2S
Source: Sci Rep. 2015 Jun 18;5:11040. doi: 10.1038/srep11040 (PMC4471901; doi:10.1038/srep11040)
Supplement: Supplementary Information [file srep11040-s1.doc]

Supplementary Materials:

Porous Au-embedded WO_3_ Nanowire Structure for Efficient Detection of CH_4_ and H_2_S

Nguyen Minh Vuong*^1,2^, Dojin Kim*^1^, Hyojin Kim^1^

^1^Department of Materials Science and Engineering, ChungnamNationalUniversity, Daejeon, 305-764 Republic of Korea

^2^Department of Physics, QuyNhon University, 170 An Duong Vuong, QuyNhon, BinhDinh, Vietnam

**Corresponding authors**: Dojin Kim, Tel: +82-42-821-6639; Fax: +82-42-823-7648, E-mail: [dojin@cnu.ac.kr](mailto:dojin@cnu.ac.kr). Nguyen Minh Vuong: E-mail: [nmvuongk23@gmail.com](mailto:nmvuongk23@gmail.com)

**1. Fabrication of sensor structures**

**
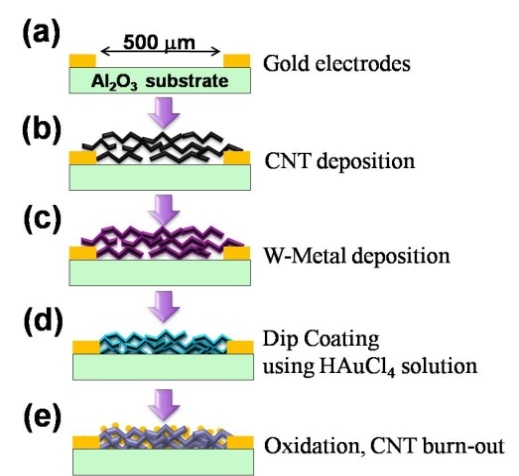
**

**FigureS1**. Flowchart of the fabrication process for Au-embedded WO_3_ porous nanowire structures using a highly porous SWCNT template.

**2. Gas sensing property measurement**

**
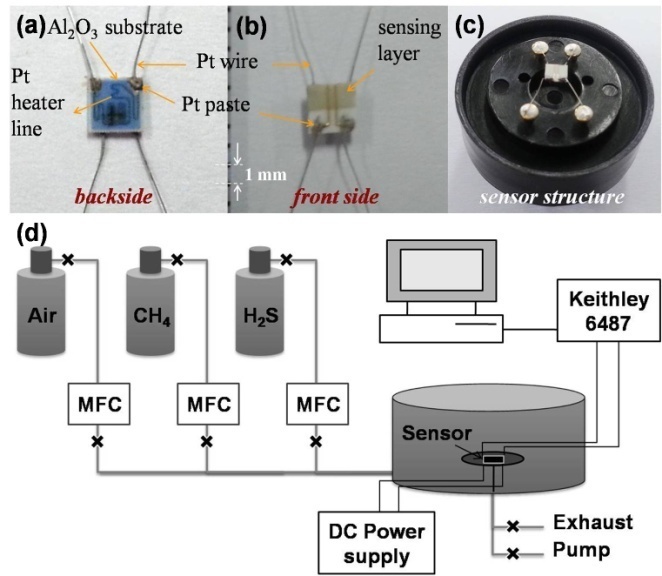
**

**FigureS2**.The images of (a) the heater in the back side, (b) sensorin the front side of the substrate, and (c) the packaged sensor. (d) A schematic diagram of the gas-sensing apparatus.

**3. Morphology of Au50-WO_3_ structure compared with WO_3_ thin film**

**
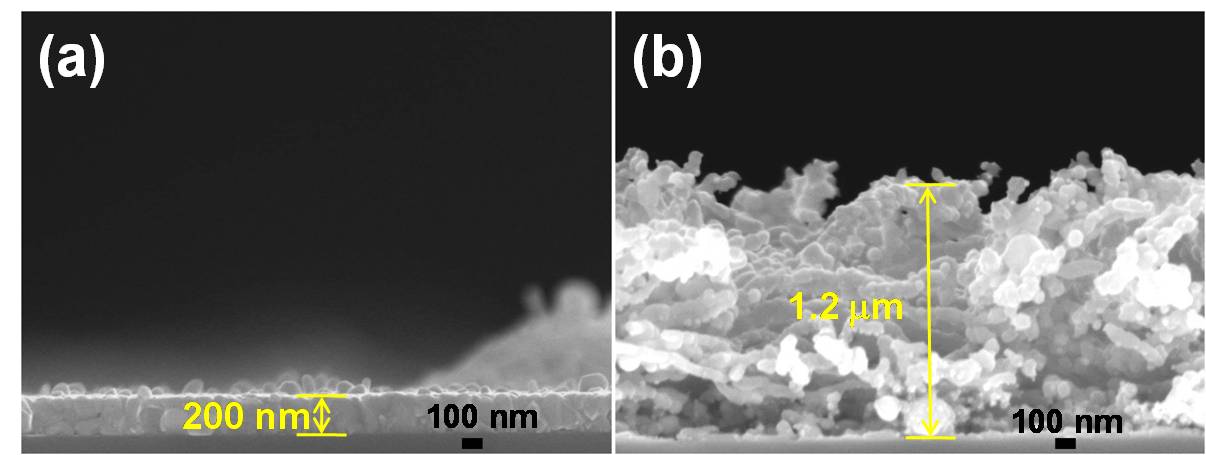
**

**Figure S3**. SEM cross-section of (a) the WO_3_ thin film on a planar substrate and (b) the Au50-WO_3_ nanowire structure fabricatedon the SWCNT template. They were fabricated with the same sputtering time, and therefore, the apparent great thickness in (b) reveals the high porosity in the structure.

**4. Selectivity of Au50-WO_3_ nanowire based sensor**

**
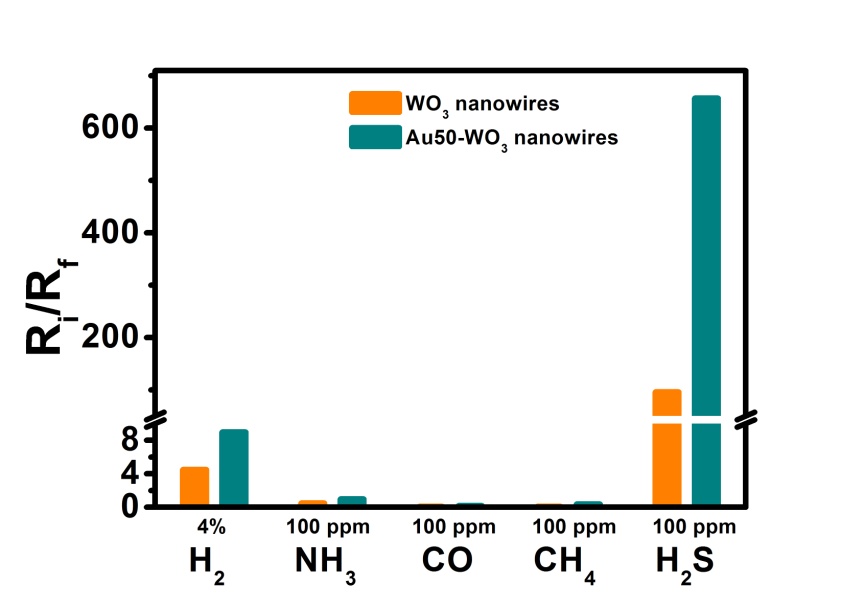
**

**Figure S4**. Selectivity histogram of pureWO_3_ and Au-embedded WO_3_ nanowire sensors.The responses were measured at an operating temperature of 291 °C for 100 ppm of the gases except H_2_ (4%).
